# EEG—Single-Channel Envelope Synchronisation and Classification for Seizure Detection and Prediction

**DOI:** 10.3390/brainsci11040516

**Published:** 2021-04-19

**Authors:** James Brian Romaine, Mario Pereira Martín, José Ramón Salvador Ortiz, José María Manzano Crespo

**Affiliations:** Departamento Ingenería, Universidad Loyola Andalucía, Dos Hermanas, 41704 Seville, Spain; mpereira@uloyola.es (M.P.M.); jrsalvador@uloyola.es (J.R.S.O.); jmanzano@uloyola.es (J.M.M.C.)

**Keywords:** epilepsy, synchronisation, envelope, DSP, hilbert transform, detection, Alzheimer disease, Parkinsons disease, prediction

## Abstract

This paper tackles the complex issue of detecting and classifying epileptic seizures whilst maintaining the total calculations at a minimum. Where many systems depend on the coupling between multiple sources, leading to hundreds of combinations of electrodes, our method calculates the instantaneous phase between non-identical upper and lower envelopes of a single-electroencephalography channel reducing the workload to the total number of electrode points. From over 600 h of simulations, our method shows a sensitivity and specificity of 100% for high false-positive rates and 83% and 75%, respectively, for moderate to low false positive rates, which compares well to both single- and multi-channel-based methods. Furthermore, pre-ictal variations in synchronisation were detected in over 90% of patients implying a possible prediction system.

## 1. Introduction

Synchronisation has been a fundamental area of study in recent decades and has been linked with many different types of disorders and diseases, such as Alzheimer Disease (AD), Parkinsons Disease (PD) and Epilepsy (EP). Synchronisation is usually obtained from a pair of sources such as from Electroencephalography (EEG), which is a multi-channel recording system of the brain’s electrical field potential, and made up of billions of Action Potentials (AP). The activity is captured using special electrodes organised into specific patterns attached to the surface of the head. The resulting signal is the difference between two electrode positions, one of which is a reference. During seizure onset neurons tend to increase rhythmic behaviour, resulting in non stationary patterns of multiple frequencies. EEG has several benefits over intracranial data recording, such as a reduction in artefacts due to spatial averaging [1]. Furthermore, it has been show that certain frequencies are responsible for certain tasks and physiological states; therefore, EEG is usually separated into multiple frequency bands, such as, infraslow<0.2 Hz, δ = 0.2–3.5 Hz, θ = 4–7.5 Hz, α,μ = 8–13 Hz, β = 14–30 Hz and γ = 30–90 Hz. For example, during sleep, the most prominent band is δ and θ, whilst the awake and alert band is considered to be β [2].

Synchronisation has proven to be an effective tool in the detection of various neurological disorders (ND). For example, in AD, synchronisation has been observed to increase in the θ and δ bands whilst, conversely, reducing in both the α and β bands at resting state. Using a synchronisation likelihood (SL) measure, it was determined that these bands decreased in synchronisation over both long- and short-range recording sites [3,4,5]. However, in PD, there is evidence to suggest that short sharp bursts of increased β activity could be responsible for tremors [6]. EP, on the other hand, is a well-studied neurological disorder with overwhelming evidence suggesting that seizures occur due to hyper-synchronous networks. High-frequency oscillations in the γ and β band were observed in EEG both during the ictal and pre-ictal periods [7]. Furthermore, the β band is of interest for its possible seizure prediction purposes, where synchronisation spikes were detected during pre-ictal periods and a synchronous activity was reduced during the ictal event [8]. Conversely, a decrease in synchronisation was proposed in [9], who examined the synchronous activity of eight patients finding a pre-ictal reduction in the β band in 77% of seizures.

EP is one of the world’s most common neurological disorders, defined by the recurrence of two or more unprovoked seizures. In fact, the incidence of EP is estimated at 61.44 per 100,000 with 95% confidence and affects all ages and races. EP can be caused by several factors, such as environmental factors, or after an attack on the central nervous system; however, in many cases the causes is unknown [10]. Although EP is common, it can be treated successfully in nearly 70% of patients using anti-epileptic medications; this leaves 30% without any possible care [11]. EP can be related to other diseases and disorders; however, Rett syndrome (RTT) is a particularly large contributor. RTT is an X-based neuro-development disorder which exists in females with an incidence rate of approximately 1 in every 10 to 20 thousand. It was estimated that between 60 and 80% of patients with RTT experience epileptic episodes [12].

Detection of synchronisation is a computationally intense process, as multiple channel methods need to calculate a measure between at least two distinct electrode positions. A 10–20 system consists of 21 electrodes, leading to total brain coverage of 210 calculations, as in [13]. In many cases, especially in implantable devices such as [14,15], it is not possible to achieve full motorisation, leading to data gaps. This, in turn, could lead to missed seizures. Single-channel methods, however, reduce the computation complexity to 21 electrodes for the 10–20 system. This is obviously more suitable for implantable devices; however, they struggle in terms of performance metrics [16]. Further examples can be found in [17], where several data acquisition methods for human brain activity can be found, along with analysis and classification of eeg signals for brain–computer interfaces.

In this manuscript, the authors intend to use the highly accurate multi-channel synchronisation method on decomposed single channels, gaining accuracy and sensitivity over other single-channel methods whilst maintaining low computational coverage. This would encourage hardware reduction, making it suitable for implantable devices.

The following paper is organised into the following sections. In Section 2, we introduce the concept behind phase synchronisation and fundamental digital signal-processing (DSP) techniques for phase extraction. In Section 3, we introduce detection methods for epilepsy and common epidemiologistic measures. Section 4 introduces our envelope-based synchronisation approach experimental methods and classification procedure. Section 5 introduces the materials and methods. Section 6 discusses the obtained results and, finally, Section 7 concludes the findings.

The main contributions of this work include the design and implementation of a novel digital signal processing (DSP) technique for the detection and prediction of epileptic seizures using single-channel synchronisation. Furthermore, a novel method to classify and verify seizures is introduced, based on an adjustable binary classifier.

## 2. Phase Synchronisation

### 2.1. Concept

In non-disturbed and noise-free systems, synchronisation is usually described as the phase locking between instantaneous phase angles, such that $n\cdot \phi_{s1}-m\cdot \phi_{s2}=const$, where $\phi_{s1}$ and $\phi_{s2}$ are the instantaneous phase angles of source 1 and source 2, respectively. m and n are integer-based weights which need adjusting based on the source of the oscillating system. However, in the case of non stationary signals, such as quasi-periodic signals, the phase-locking condition should be considered as $|n\cdot \phi_{s1}-m\cdot \phi_{s2}-\omega |<const$, where ω is some average phase shift. This condition indicates that, although the rate of change is not completely constant, it should reside (fluctuate) around a common constant [18]. In many cases of phase-locking, the values *m* and *n* are usually set to a fixed 1:1 ratio. This is especially true if the signals originate from the same source, such as the brain.

### 2.2. Hilbert Transform

The Hilbert transform (HT) is a common signal-processing technique used in the evaluation of phase synchronisation. The HT creates a 90${}^{\circ }$ phase-shifted version of the original signal s(t), which is projected onto the imaginary plane. This allows for easy instantaneous phase extraction [19]:

(1) $$\tilde{s}\left(t\right)=\frac{1}{\pi }\int_{-\infty }^{+\infty }\frac{s(\tau )}{t-\tau }\cdot d\tau$$

where the integral is taken in the sense of Cauchy principal value for t=τ .

### 2.3. Analytical Signal

The analytical signal is a representation of complex analogue signal s(t), which is composed of both the real and imaginary parts $s_{R}\left(t\right),s_{I}\left(t\right)$. The relationship between the two parts is given as $s\left(t\right)=s_{R}\left(t\right)+j\cdot s_{I}\left(t\right)$, where $j=\sqrt{-1}$. Likewise, a complex number can also be represented in terms of its magnitude *a* and phase angle θ, such that s(t)=aexp(jθ(t)). From Equation (1), the analytical signal can be defined as

(2) $$x\left(t\right)=s\left(t\right)+j\cdot \tilde{s}\left(t\right),$$

where $\tilde{s}\left(t\right)$, is the HT of s(t).

Given the phase-magnitude representation, it is possible to extract the instantaneous magnitude of the signal, defined as

(3) $$a\left(t\right)=\sqrt{s^{2}\left(t\right)+{\tilde{s}}^{2}\left(t\right)}.$$

a(t), is often referred to as the complex analytic envelope of s(t), whereas its module |a(t)| is referred to simply as the analytic envelope of s(t). The instantaneous phase angle of the signal is defined as

(4) $$\varphi_{s}\left(t\right)=\arctan\frac{\tilde{s}\left(t\right)}{s(t)}$$

### 2.4. Phase Locking Value

The Phase-Locking Value (PLV) is a method for quantifying the relationship between a specific set of phase angles. This method is usually adopted along with the HT and wavelet transform (WT) methods for calculating phase synchronisation, and converts windows of instantaneous phase angles into unit vectors. In the case where all of the unit vectors point in the same direction, the PLV will result in a value of 1, indicating maximum synchronisation. Contrarily, if all of the unit vectors point in different directions, the PLV will be 0, which signifies minimum synchronisation. The PLV is defined as

(5) $$\;PLV=|\frac{1}{N}\sum_{K=0}^{N-1}e^{j\phi_{k}\left(t\right)}|=\frac{1}{N}\cdot \sqrt{{\left[\sum_{K=0}^{N-1}\cos\left(\Delta \phi_{k}\right)\right]}^{2}+[\sum_{K=0}^{N-1}\sin\left(\Delta \phi_{k}\right){]}^{2}}$$

where $\Delta \varphi_{k}=\left(n\cdot \varphi_{eu}-m\cdot \varphi_{el}\right)$ are the relative phase angles between two sources taken over a specific window of *N* samples. In our case, the unit vectors are constructed as the relationship between the instantaneous phase angles of the upper and lower interpolated envelopes [20,21].

## 3. Epilepsy Detection

At present, there are dozens of methods which claim the successful detection of epileptic seizures in the time, wavelet and frequency domains, which act on either a single channel or multiple channels. Table 1 shows some examples of epilepsy detection or prediction methods. For single-channel methods, in the time domain, many features can be combined using feature integrators, such as in [22], where they combine the noise levels, entropy, Lyapunov Exponent and others in order to improve the overall sensitivity, which can be greater than 90%. Other methods in the time domain rely on statistical analysis and probability forecasting, such as in [23]. However, these methods usually need to be multi-feature, which is reflected in the sensitivity of 60%. Much of the research in the area of epilepsy detection and prediction is focused on the frequency and wavlelet domains. One such example of frequency analysis is [24], where the authors use the intrinsic mode function from an empirical mode decomposition (EMD) to calculate the instantaneous area, reaching 90% sensitivity with no classifier; however, the FPR rate was not stated.

### Performance Indexes

Performance indexes form part of epidemiology, which is the study of distributions and determinants of health events; several import diagnostics are included, which should be used to assess the potential liability of a test. Below, we will show four of the most common; however, many more do exist. The diagnostic tests rely on several key measurements. To begin, a true positive (TP) indicates when a seizure was correctly detected by the test. A false positive (FP) indicates when that a seizure was wrongly detected by the test. A true negative (TN) occurs when the test correctly identifies no seizure. Finally, a false negative (FN) occurs when a the test incorrectly declares no seizure [28].

The sensitivity is the fraction of people that the test will correctly identify as having seizures and is calculated as

(6) $$Sensitivity=\frac{\sum TP}{\sum TP+\sum FN}$$

The specifitiy is the fraction of people without seizures which the test will correctly identify as not having seizures and calculated as

(7) $$Specifity=\frac{\sum TN}{\sum TN+\sum FP}$$

The accuracy is calculated as

(8) $$Accuracy=\frac{\sum TP+\sum TN}{\sum TN+\sum FP+\sum FN+\sum TP}$$

## 4. Envelope-Based Phase Synchronisation and Classification

The envelope of a signal can be thought of as the imaginary curve which encompasses the upper and lower extremes of any given signal. Several key techniques can be used to find the envelopes, such as the use of the analytical signal, as described in (3). However, the analytical approach leaves us with an identical upper and lower envelope, which offers little information on the rapidly changing phase angle relationship between upper and lower. One interesting method is the use of peak detection, where the local maxima and minima of the signal are found and connected via interpolation. Common interpolation methods include the use of polynomial and cubic splines. In our case, we use a custom, simplified and hardware friendly maxima and minma detection scheme based on zero crossings and sample constraints as follows:

- For a given signal. a sample is defined as $S_{i}$, where *S* is the signal to which the sample belongs and *i* is the sample position;
- If $S_{i-1}>0$, then consider that we are in the positive half wave, and if $S_{i}>S_{i-1}$, then consider that there is a new maximum to be detected. To reduce artefacts and noise, we add a 20-point constraint, such that the current maximum value must be greater than 20 samples after the previous maximum. If the condition is met the new maximum will be stored;
- For the negative half, we check if $S_{i-1}<0$ and $S_{i}<S_{i-1}$, then consider a new minimum to be detected and store the minimum value if 20-point condition is met;
- Repeat the process until 2·N values have been collected.

**Figure 1:**
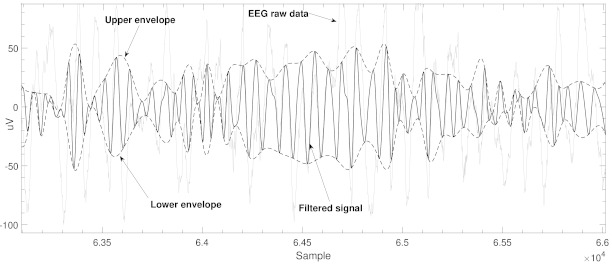
This figure shows an example of the original EEG raw signal, β filtered signal and the respective envelopes using a 20-point spline interpolated max,min schema.

The *N* points are then connected using a cubic spline interpolation. Figure 1 shows an example of the envelopes alongside the original and filtered signal. The varying frequencies of each envelope, in turn, affect the instantaneous phase angles [29,30].

### Classification

**Figure 2:**
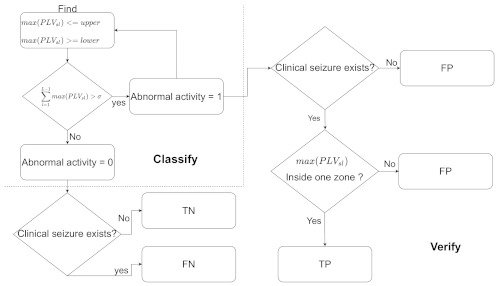
This figure shows an overview of the classification and verification process.

The classification of seizures can be seen in Figure 2 and can be separated into two main steps. First and foremost, we must identify any type of abnormal activity which could correspond with a potential seizure. Secondly, a threshold should be placed on the abnormal activity (to be defined later). To classify the PLV results, we design a two-stage binary classifier, which results in a logical True if σ% of the maximum PLV values fall within a given upper and lower boundary and a logical False otherwise.

First, the PLV data are sifted to find the maximum result for each individual EEG signal; once found, the corresponding vector locations are stored in a new vector $V_{p}$, where each location corresponds directly to 1 s of time.

Next, the boundaries are built with a ρ% tolerance such that

(9) $$upper=\mu +\mu \cdot \frac{\rho }{100}$$

(10) $$lower=\mu -\mu \cdot \frac{\rho }{100}$$

where μ is the median of vector $V_{p}$.

The second stage is to appropriately set ρ such that the abnormal activity is classified as a seizure or not. The threshold is set as the number of the maximum PLV values which fall within the boundaries, such that

(11) $$abnormal\;activity=\left\{\begin{array}{cc} 1 & \mathrm{if}\;\sum_{l=1}^{L-1}max\left(PLV_{sl}\right)>\sigma \\ 0 & \mathrm{otherwise} \end{array}\right.$$

where σ is the maximum PLV values.

**Figure 3:**
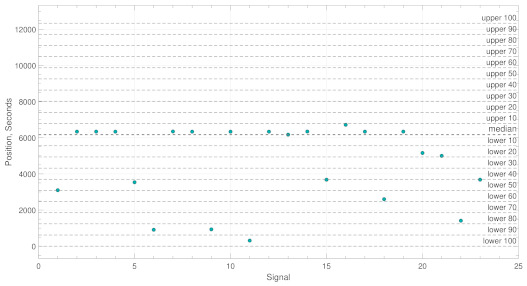
This figure shows an example of a logical true classification process used to identify abnormal activity. The y-values represent the position of the maximum PLV values in seconds and the boundaries represent the constraint ρ. In this case, abnormal activity is found.

*Example A:*Figure 3 shows an example of the the time positions of the maximum PLV values for a patient who suffered an ictal event. In this case, it can be noted that 14 out of 23 (just over 60%) maximum PLV values fall within the boundary ρ=20; this increases to 18 out of 23 (just under 80%) for ρ=50. For both ρ=20 and ρ=50, a value of σ<19 would result in abnormal activity.

**Figure 4:**
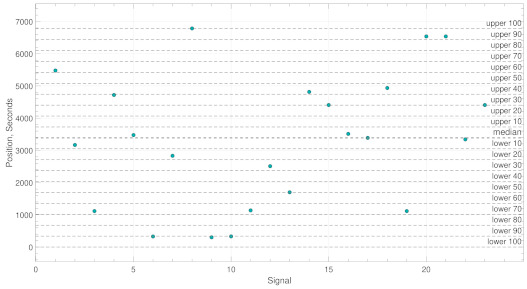
This figure shows an example of a logical flase classification process used to identify abnormal activity. The y-values represent the position of the maximum PLV values in seconds and the boundaries represent the constraint ρ. In this case, no abnormal activity is found.

*Example B:*Figure 4 we note that if just 9 out of 23 (39%) fall within the boundaries with ρ=50 the classifier would conclude no abnormal activity.

The classification is finalised by verifying this abnormal activity given by the binary classifer with respect to the clinically diagnosed seizures available in the chosen dataset. A TP is set as true by calculating how many of the maximum PLV values which fall under the abnormal activity condition in (11) fall within a designated zone, where the dedicated zones are separated into seven distinct sections, as described in Table 2. These zones allow us to analyse the preictal portion of the seizures as possible prediction biomarkers.

An FP is set if abnormal activity was detected but no clinical seizure is present in any zone. Finally, a TN is set if no abnormal activity was detected and no clinical seizure exists.

In our Example B for ρ=20 and σ=10, the classifier would produce a TN, and in Example A, the classifier would produce a TP.

## 5. Materials and Methods

For the experimental setup, the CHB-MIT database from Physionet was used [27]. This database consists of 916 h of continuous scalp EEG data for 22 subjects (5 males, ages 3–22 and 17 females, ages 1.5–19). All signals were sampled at 256 samples per second with 16-bit resolution and captured using scalp electrodes organised into the international 10–20 system of EEG electrode positions. These records include 198 seizures in total. Please find information on patients used in this manuscript in Table A1, including age and sex of the patients.

### Main Setup

**Figure 5:**
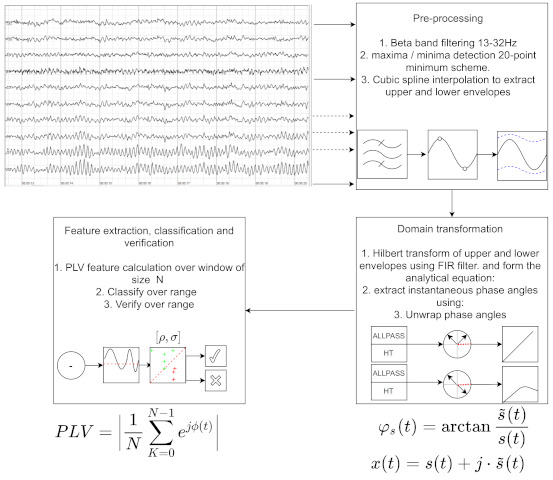
This figure shows an example overview of the full DSP process including the pre-processing, domain changes and feature extraction.

The main setup consists of several key steps which can be seen in Figure 5 and are as follows:

1. Filter signals
  - The EEG signals are multi-frequency signals where 0<f<150 Hz, making them non-stationary signals. As explained in Section 2.2, the HT only makes sense over a small finite frequency range. Furthermore, as explained in Section 3 seizures generally occur in specific frequency bands which correspond to different human physical states. Therefore, we introduced a pre-filtering stage into the β∼ 12–30 Hz frequency band. This was achieved using band-pass equiripple FIR zero-phase filters and the specific band was chosen as it corresponds to the awake and alert state.
2. Calculate HT
  - The HT is calculated in four steps. Firstly, the the fast Fourier transform (FFT) is calculated for the input signal. Next, a new vector h(i) is formed such that h(i)=1 for i=1,(n/2)+1, h(i)=2 for i=2,3,...,(n/2) and h(i)=0 for i=(n/2)+2,...,n. The inverse FFT is then taken from the product of h(i) and the input signal.
3. Calculate upper and lower envelopes for each of the filtered signals.
  - The upper envelope is calculated using a 20-point maxima detection scheme, as described in Section 4. These points are then interconnected using a cubic spline interpolation. The lower envelopes are calculated in the same way, but using the minima instead of the maxima.
4. Calculate phase angles
  - The phase angles are calculated according to (4). Producing the instantaneous phase angles bound between −2π to +2π. To avoid phase slippage, the angles are then unwrapped. Whenever the jump between consecutive angles is greater than or equal to π radians, unwrap shifts the angles by adding multiples of ±2π until the jump is less than π.
5. Calculate PLV
  - The PLV values for each signal are calculated according to (5). In all experiments, the window size *N* was set as a rectangular window with a size equal to the sampling rate of the signal multiplied by ten. Since the sampling rate is equal to 1 s of data, this gives a window size of 10 s. The window is lid across the data at in increments of N/2, leading to a 50% data overlap.
6. Classify
  - Next, abnormal activity is classified as described in Section 4.
7. Calculate sensitivity, specifity and accuracy per (6)–(8)

## 6. Results

Over 600 h of simulations were run on patients 3, 5, 8 and 10 over a range of 10% to 100% in steps of 10% for both σ and ρ. ρ is held fixed whilst σ scans the range. Once finished, ρ is allowed to increment one position and continues until the full range is complete. This provides us with a map of the best classification variables for each patient and allows us to analyse the metrics in Section 3.

**Figure 6:**
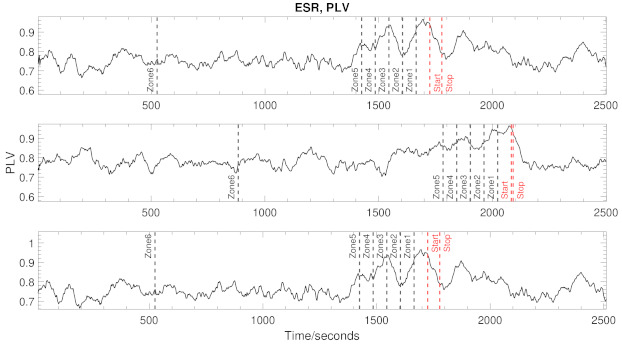
This figure shows the the PLV results of signal 2 from 3 separate patients, all with a clinically diagnosed seizure. The zones are pre-ictal definition sections, as explained in Section 4.

Figure 6 shows an example of the raw PLV results from an hour long recording of three patients using the envelope synchronisation method. In each case, a single, clinically diagnosed seizure is present in the range marked *start*, *stop*. In all three patients, we can see that the peak synchronisation between envelopes happens before (in zone 1) the clinical seizure occurs, and multiple peaks can also be found in zones 3 and zones 4.

**Figure 7:**
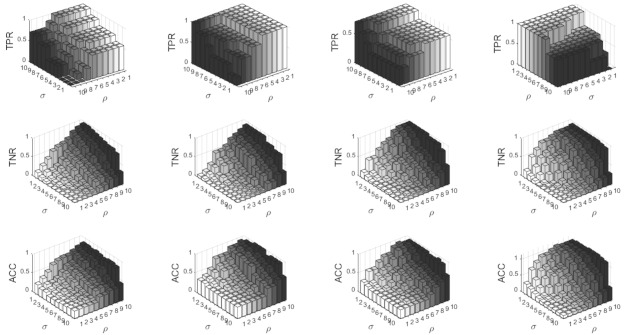
This figure shows the sensitivity, specifity and accuracy results for 4 patients 3, 5, 8 and 10 over a range of σ = 10–100% and ρ=10%−100%. From top to bottom, sensitivity, specifity and accuracy.

Figure 7, shows the sensitivity, specifity and accuracy results for four of the patients. In the case of sensitivity, the detection method works extremely well, producing a maximum sensitivity of 100% for high σ and low values of ρ. The sensitivity diminishes as ρ increases and σ decreases, as the boundary becomes wider and artefacts are more inclined to be detected. This ultimately increases the FPR. As for the specifity, the results are conversely related to the TPR, increasing with increasing ρ and decreasing σ, and ultimately produce the highest possible specificty rate of 100% in all patients. Finally, the accuracy is high for all patients for increasing ρ and decreasing σ and reaches values close to 100%. Nevertheless, σ seems less relevant in this case, where the boundaries have the largest effect.

Another interesting observation is related to the boundaries. From Figure 7, for patient 3,5 and 8, the best values of sensitivity, specifity and accuracy fall in zone 1, which is a dedicated pre-ictal zone. This indicates that it is possible to detect upcoming seizures via an increase in synchronisation pre-ictal. Table 3, shows the sensitivity, specifity and accuracy of the four patients in all of the predefined zones. Here, we can note that, apart from zone 1 and zone 2, the other zones produce little sensitvity but high specifity. In the case of patient 5, zone 6 produced a high accuracy of 87%.

In a real life, implementation of the classification process would be run on the patient to build a look-up-table of the best values of σ and ρ.

**Figure 8:**
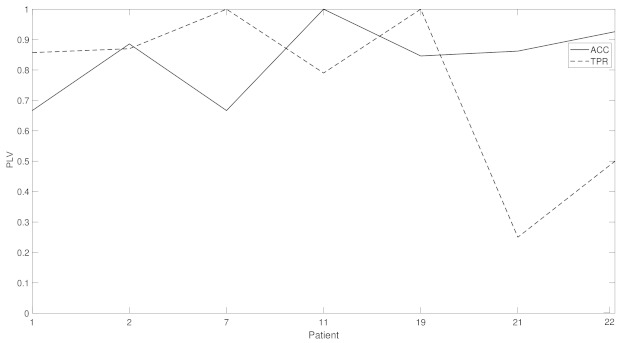
This figure shows the true positive rate (TPR) and accuracy (ACC) for patients 1, 2, 7, 11, 19, 21 and 22 for values σ=7 and ρ=8.

Figure 8 shows the sensitivity and accuracy for seven other patients, this time with a fixed σ=7 and ρ=8. A mean accuracy of over 83% and of 75% is measured.

## 7. Conclusions

In this paper, the authors have shown that the upper and lower envelopes of filtered EEG signals can be used to detect epileptic episodes with high sensitivity and specificity, and an accuracy of up to 100%, with varying classification values σ and ρ, for four patients, which compares to the best single- and multi-channel methods to date. A further seven patients were tested for fixed values of σ=7 and ρ=8, producing an average accuracy of 83% and 75%, respectively, confirming its validity over various patients . An interesting fact is that this method also allowed for the detection of upcoming seizures in the pre-ictal regions, where three of the four patients tested in the classifier showed maximum sensitivity, accuracy and specificity during the fist 60 s prior to the ictal event . The low number of channels necessary for detection makes the system ideal for implementation as a medical prosthesis.

## Figures and Tables

**Table 1:** This table shows some examples of epilepsy detection methods.

	Method	Database	Domain	Frame Length	Classifier	Sen, Spec, Acc
[22]	Time analysis correlation entropy	Freiburg database [25]	Time	10s	None	Sen 75% to >90%
[23]	Time analysis Probability TF	Unknown 8-patients	Time	30s	None	Sen 60%
[2]	frequency analysis phase entropy	Unknown	Frequency	23s	SVM	Acc 98%
[26]	Wavelet analysis Coherence	CHB-MIT database [27]	Wavelet	60s	SVM	Sen 52% to 74%
[24]	EMD analysis correlation entropy	Freiburg database [25]	Frequency	15s	None	Sen, 90% Spec, 89%

**Table 2:** This table shows the specified pre-ictal zones as defined.

Zone	Start	Stop
zone1	clinical start	clinical end
zone2	clinical start—60 sec	clinical start
zone3	zone2 start—60 sec	zone 2 start
zone4	zone3 start—60 sec	zone3 start
zone5	zone4 start—60 sec	zone4 start
zone6	zone5 start—60 sec	zone5 start
zone7	zone6 start—1000 sec	zone6 start

**Table 3:** This shows the mean second-best results for sensitivity, specificity and accuracy, respectively, in each zone over all values of ρ and σ.

Sensitivity, Specificity, Accuracy							
Patient	Seizure Zone	Zone 1	Zone 2	Zone 3	Zone 4	Zone 5	Zone 6
3	0.4,0.8,0.74	best	0.11,0.65,0.74	0,0.8,0.23	0,0.81,0.1	0.12,0.76,0	0,0.74,0
5	0.9,0.86,1	best	0,0.86,1	0,1,0.9	0.16,1,0.9	0,1,0.87	0.3,1,0.87
8	0.2,0.8,0.83	best	0.2,0.48,0.83	0,0.78,0.52	0,0.91,0	0,0.77,0.04	0.32,0.75,0.28
10	best	0.14,0.08,0.79	0,0.48,0.79	0,0.9,0	0,0.8,0	0,0.78,0	0.2,0.78,0

## Data Availability

CHB-MIT EEG dataset Open source: https://physionet.org/content/chbmit/1.0.0/, accessed on 21-March-2021.

## Abbreviations

The following abbreviations are used in this manuscript:

PLV	Phase Locking Value
PD	Parkinsons Disease
AD	Alzhimers Disease
EP	Epilepsy
EEG	Electroencephalogram
TP	True Positive
FN	False Negative
TN	True Negative
FP	False Positive
HT	Hilbert Transform
DSP	Digital Signal Processing
FPR	false positive rate
Sen	Sensitivity
Spe	Specifity
Acc	Accuracy
TPR	True Positive Rate
WT	Wavelet Transform

## Appendix A

**Table A1:** This table shows patient information used in this manuscript.

Patient	Age (Years)	Gender (F/M)
1	11	F
2	11	M
3	14	F
5	7	F
7	14.5	F
8	3.5	M
10	3	M
11	12	F
19	19	F
21	13	F
22	9	F
